# Heterologous expression of *lcc1 *gene from *Trametes trogii *in *Pichia pastoris *and characterization of the recombinant enzyme

**DOI:** 10.1186/1475-2859-5-31

**Published:** 2006-10-12

**Authors:** Maria Chiara Colao, Stefania Lupino, Anna Maria Garzillo, Vincenzo Buonocore, Maurizio Ruzzi

**Affiliations:** 1 Department of Agrobiology and Agrochemistry, Tuscia University, Via C. de Lellis s.n.c., I-01100 Viterbo, Italy

## Abstract

**Background:**

Fungal laccases are useful enzymes for industrial applications; they exhibit broad substrate specificity and thus are able to oxidize a variety of xenobiotic compounds including chlorinated phenolics, synthetic dyes, pesticides and polycyclic aromatic hydrocarbons. Unfortunately, the biotechnological exploitation of laccases can be hampered by the difficulties concerning the enzyme production by the native hosts.

**Results:**

In order to obtain a simple and efficient source of laccase, the *lcc1 *cDNA isolated from the white-rot fungus *Trametes trogii *has been successfully expressed in the methylotrophic yeast *Pichia pastoris *under the control of the methanol induced alcohol oxidase promoter P_AOX1_. The recombinant Lcc1 was produced as a secreted protein with the native N-terminal prepropeptide for signal trafficking, and thus easily recovered from the culture medium. At the 1-liter scale, as calculated on the basis of the specific activity, the recombinant protein was produced at a yield of 17 mg/l. The highest production level obtained in fed-batch culture was 2520 U/l, corresponding to a specific productivity of 31.5 U/g biomass.

The purified recombinant laccase exhibited a behaviour similar to the main laccase produced by *T. trogii*. Lcc1 showed high activity in the presence of organic solvents and a high decolourization capacity towards azo, triarylmethane, indigo carmine and anthraquinonic dyes, that could be significantly enhanced in the presence of the redox mediators 1-hydroxybenzotriazole and violuric acid.

**Conclusion:**

Heterologous expression of *T. trogii *laccase *lcc1 *in the methylotrophic yeast *P. pastoris *was successfully achieved. The biochemical and kinetic characterization of the recombinant protein suggests potential technological applications for this enzyme.

## Background

Laccases (EC 1.10.3.2) belong to the family of the multi-copper oxidases and catalyze the one-oxidation of a wide variety of substrates, especially phenolic compounds and aromatic amines. One electron at a time is removed from the substrate by a type-1 blue copper ion and is transferred to a trinuclear copper cluster; molecular oxygen is used as electron acceptor. The substrate loses a single electron and usually forms a free radical that may undergo further laccase-catalysed oxidation or non enzymatic reactions including hydratation, disproportionation and polymerization [1]. Laccases have been described in fungi, plants and bacteria; fungal laccases have been suggested to participate in lignin depolymerization by wood-rotting and plant pathogens. Additional physiological functions of fungal laccases in pigmentation, fruiting body formation and pathogenicity expression have also been suggested [2].

Because of their low substrate specificity, laccases may have a number of biotechnological applications, including pulp delignification, detoxification of recalcitrant biochemicals, polycyclic aromatic hydrocarbons degradation, wastewater and soil bioremediation, and organic synthesis [3]. In particular, recent studies have shown that fungal laccases can decolourize and detoxify industrial dyes *in vitro *[4-6] and that the substrate specificity of the enzyme can be broadened in the presence of redox mediators [7].

Enzyme production on industrial scale is feasible when the protein is formed at high levels and the producing organism can be cultivated in a large-scale fermentation. Thus, the genes for many industrially important enzymes have been inserted in a heterologous host such as filamentous fungi and yeasts. A frequently used expression organism is the methylotrophic yeast *Pichia pastoris *that can grow on methanol as sole carbon and energy source [8]. The recombinant proteins can be expressed under the control of a strong tightly regulated promoter, the methanol induced alcohol oxidase P_AOX1_. *P. pastoris *has the potential for high expression levels, efficient secretion of extracellular proteins, post translational modifications, such as glycosylation, and growth at high cell densities on defined minimal medium. Laccase genes from *T. versicolor *[9,10], *Pycnoporus cinnabarinus *[11], *Pleurotus sajor-caju *[12] and *Fome lignosus *[13] have been expressed in *P. pastoris *indicating the suitability of this system for laccase production. Recently, a number of laccase genes has been also expressed in the filamentous fungi *Trichoderma reseei *[14] and *Aspergillus *[15,16]; although filamentous fungi are generally good hosts for protein secretion, they are more time consuming to work with, compared to yeast.

The white-rot fungus *Trametes trogii *201 produces a major phenol oxidase (formerly coded as PoxL3) that has been widely characterized [17,18]; the corresponding gene, *lcc1*, has been isolated and sequenced [19]. In addition, the fungus secretes in the cultural broths low levels of at least four additional laccases. Isoenzyme multiplicity is commonly observed among ligninolytic fungi although its physiological significance is not known yet. The presence of several isoforms with similar chemico-physical properties makes difficult to purify individual enzymes for analysis, a problem that can be overcome expressing the corresponding genes in a heterologous host. Moreover, the recombinant expression system may lead to the production of enzymes easy to purify and would allow protein engineering studies to construct laccases with desired properties.

In the present work the *lcc1 *cDNA from *T. trogii *was expressed in *P. pastoris*; the recombinant protein has been purified and its kinetic and chemico-physical properties have been determined and compared with those of the native enzyme. The recombinant Lcc1 was tested for its ability to operate in the presence of organic solvents and decolourize different synthetic textile dyes.

## Results

### 1. Heterologous expression of lcc1 in Pichia pastoris

Two different vectors, pHIL-D2 and pPIC9, were employed for the laccase production in the methylotrophic yeast *P. pastoris*. The *lcc1 *cDNA sequence from *T. trogii *201 was expressed in GS115 (*his4*) and SMD1168 (*his4, pep4*) strains under the control of the tightly regulated alcohol oxidase promoter (P_AOX1_) induced by methanol, using either the native signal sequence or the α-factor signal peptide from *S. cerevisiae *to direct the secretion of the recombinant protein. The enzymatic activity was detected on ABTS containing plates in the presence of methanol as inducer and sole carbon source. In these conditions, a green halo surrounding the colonies indicated the production of an active recombinant enzyme secreted in the medium. Transformants showing a deeper colour were used for the production of the recombinant protein using liquid cultures. In preliminary experiments carried out in shaken flasks, the laccase activity was found in the culture medium and no intracellular activity was detected at any time during the growth on minimal medium with methanol as a carbon source. Enzymatic assays on the liquid medium showed that the laccase activity of positive transformants was about 2 U/l after 7 days of growth; in these experiments the pH of the culture decreases to 3.0 after 24 hours from the addition of methanol (data not shown). It has been reported that the yield of recombinant proteins, such as the mouse epidermal growth factor (mEGF), can be increased using a buffered medium at pH 6.0 amended with 1% casaminoacids [20]. A significant increase in laccase production was obtained in a buffered medium by the addition of casaminoacids or alternative sources of organic nitrogen and nutritional factors, such as yeast extract, which could affect production and/or activity of extracellular proteases. The highest expression level obtained for recombinant Lcc1 was about 173 U/l, in a buffered saline medium amended with yeast extract after 7 days of growth. In the medium amended with casaminoacids, laccase production reached 100 U/l at day 4 and then decreased to 65 U/l at day 7. As already observed by Clare et al. [20] for the production of mEGF, the final level of accumulation of laccase was dependent on the composition of the medium and could be increased amending the buffered medium with an organic source of nitrogen. Moreover the native signal peptide was a more efficient secretion signal as compared to the α-factor prepro signal sequence.

The use of a proteinase A mutant strain (SMD1168), reported to be beneficial for the production of secreted recombinant proteins, had no advantage for the production of Lcc1 in shaken flask cultures when transformants were grown in a buffered medium supplemented with yeast extract; the laccase activity produced at day 7 was about 51 U/l (data not shown).

### 2. Production of recombinant laccase in bench-top fermentor

The recombinant laccase production in *P. pastoris *was optimized under controlled conditions using a fermentor cultivation. Transformants were grown at 30°C in a basal salt medium containing glycerol or methanol as a carbon source in a 2-l bench-top fermentor. The fermentation process included three different stages in which the dissolved oxygen spike method was used to devise the feeding scheme. As first, cells were grown in a batch mode using an excess of glycerol as carbon source. Following the glycerol exhaustion, a limited glycerol feed was initiated in a fed-batch mode, such that glycerol did not accumulate in the medium. The third stage of the fermentation was initiated by replacing glycerol with methanol as the carbon source.

**Figure 1:**
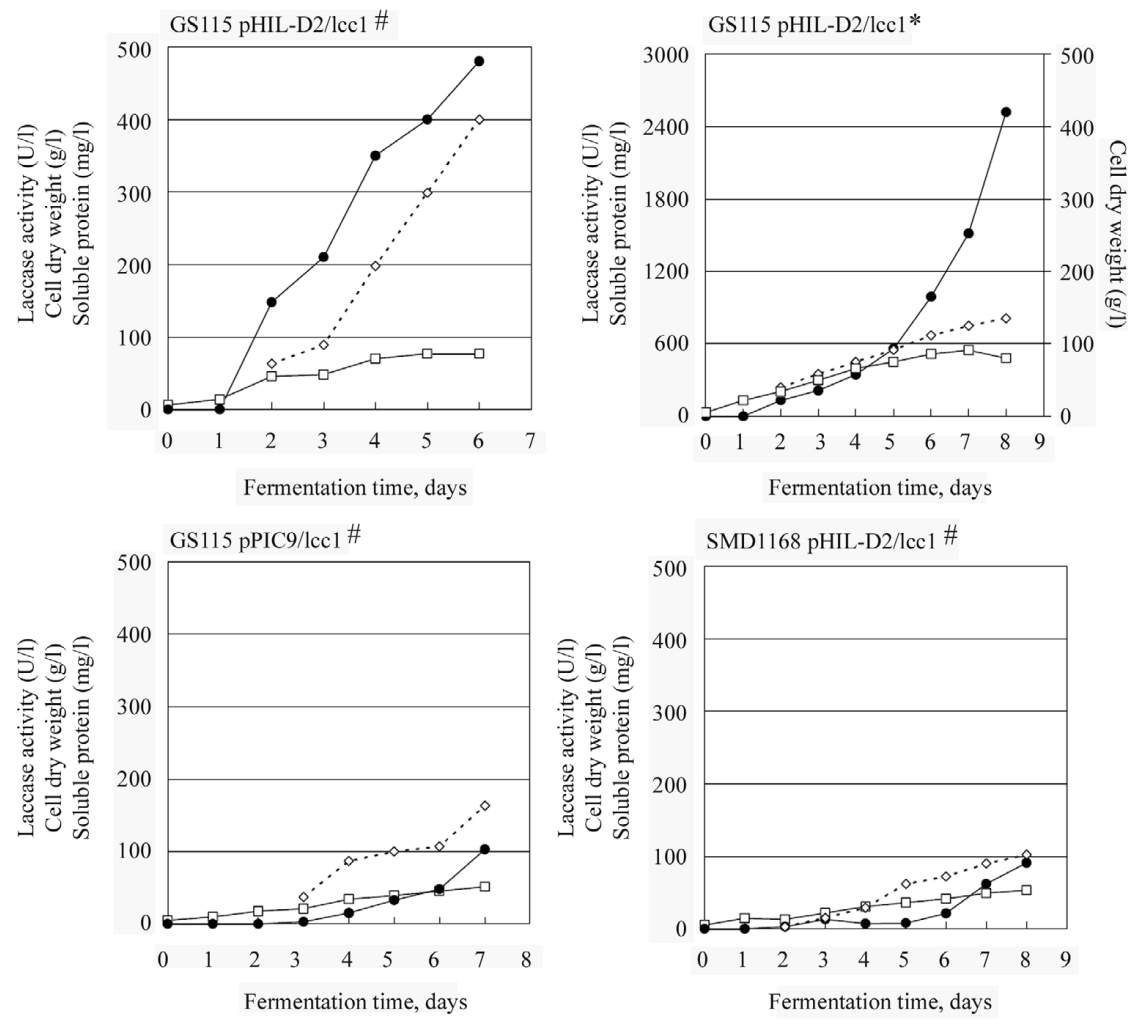
**Fermentor-based production of recombinant Lcc1 by *P. pastoris***. Different fermentation protocols (#: standard conditions, *: optimised conditions) secretion signal peptides and strains have been compared. Cell dry weight (□), laccase activity (●) and total soluble protein (◇) are reported. Data points are averages of triplicate measurements with SD within 10% of the mean.

The results obtained confirmed that the levels of laccase activity, dry cell biomass and total proteins were differently affected on the prepro sequence used to direct the secretion of the laccase as well as on the host strain used (Fig. 1). The analysis of the volumetric [U(ABTS)/l] and specific [U(ABTS)/g dry biomass] productivity of the recombinant protein showed that the laccase levels were at least 4 times higher when the native signal peptide was employed as compared to the α-factor and the use of SMD1168 had no advantage for the production of Lcc1. A volumetric productivity of 480 U/l and a specific productivity of 6.2 U/g dry biomass were obtained with the strain GS115 using the native signal peptide to drive the secretion of the recombinant Lcc1. The expression of the recombinant laccase was further improved adjusting the methanol feed and providing oxygen-enriched aeration: accurate regulation of the methanol feed is a key point to maintain the induction of the genes under the control of the AOX1 promoter and to prevent methanol accumulation at levels that were toxic to the cells. Under the tested conditions (Fig. 1), the growth phase on glycerol finished when the biomass had reached 25 g/l dry weight, and the induction phase was initiated by feeding methanol and increasing the flow rate up to 18 ml/l·h; pure oxygen was supplemented as required up to 0.2 v/v/min. A laccase activity of 2520 U/l, corresponding to 17 mg/l, was reached after 8 days at a cell dry weight of 80 g/l with a specific productivity of 31.5 U/g biomass; the total soluble protein concentration after eight days was 810 mg/l. The volumetric productivity in fermentor cultures was at least 14 times higher than in shake flasks.

### 3. Protein purification and characterization

**Figure 2:**
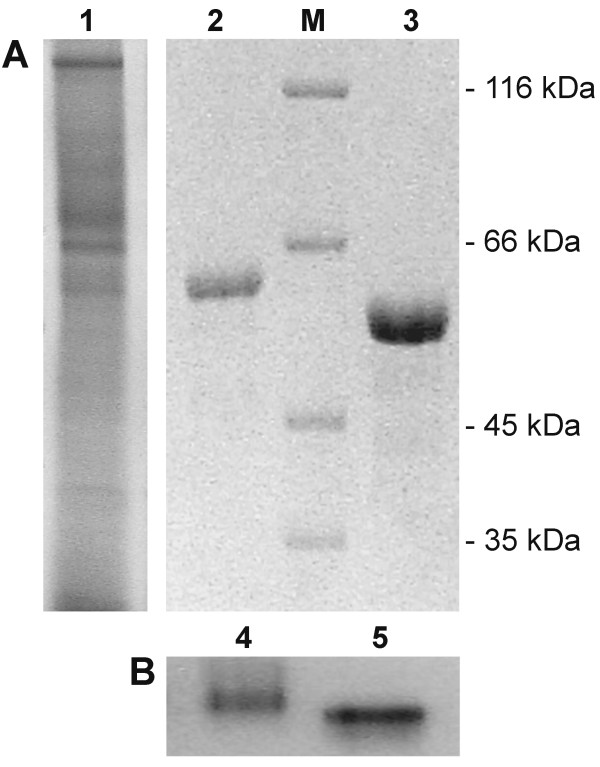
**Electrophoretic analysis of the recombinant Lcc1**. Panel A: SDS-PAGE of culture ultrafiltrate (lane 1), purified recombinant Lcc1 (lane 2) and native Lcc1 (lane 3); M, molecular weight markers. Panel B: PAGE of the recombinant (lane 4) and native (lane 5) Lcc1 stained by *p*-phenylendiamine as a substrate.

The recombinant laccase was purified from the culture medium of *P. pastoris *GS115 (pHIL-D2/*lcc1*) by three chromatographic steps (Table 1). The homogeneous enzyme was obtained with an overall yield of 16% and a specific activity of 232 U/mg. The homogeneity of the purified protein was checked on both native and SDS-polyacrylamide gel electrophoresis; the enzyme showed a single band of 64 kDa (Fig. 2). The higher molecular weight of the recombinant laccase as compared with the native enzyme (60 kDa) can be ascribed to a higher level of glycosylation of the enzyme expressed by *P. pastoris*. In fact the two proteins, after treatment with endoglycosidase H, showed similar molecular mass and did not react positively to PAS staining (data not shown). The hyperglycosylation of the recombinant laccase did not affect activity, since both native and recombinant Lcc1 exhibited similar specific activity. The N-terminal sequencing of the recombinant Lcc1 showed that the native signal peptide was correctly processed.

**Table 1:** Purification of the recombinant *T. trogii *laccase encoded by *lcc1*

**Purification step**	**Volume (ml)**	**Activity (U/ml)**	**Protein (mg/ml)**	**Specific activity (U/mg)**	**Activity yield (%)**	**Purification factor**
**Culture filtrate**	670.0	0.3	0.12	2.6	100	1
**Ultrafiltrate**	14.0	14.1	1.19	11.8	95	4
**Q-Sepharose FF**	11.0	16.6	0.59	28.2	88	10
**Phenyl-Sepharose FF**	0.5	151.6	1.30	116.6	36	45
**Superdex 75**	1.0	33.0	0.14	232.4	16	89

### 4. Kinetic analysis and stability of Lcc1 to temperature and pH

**Figure 3:**
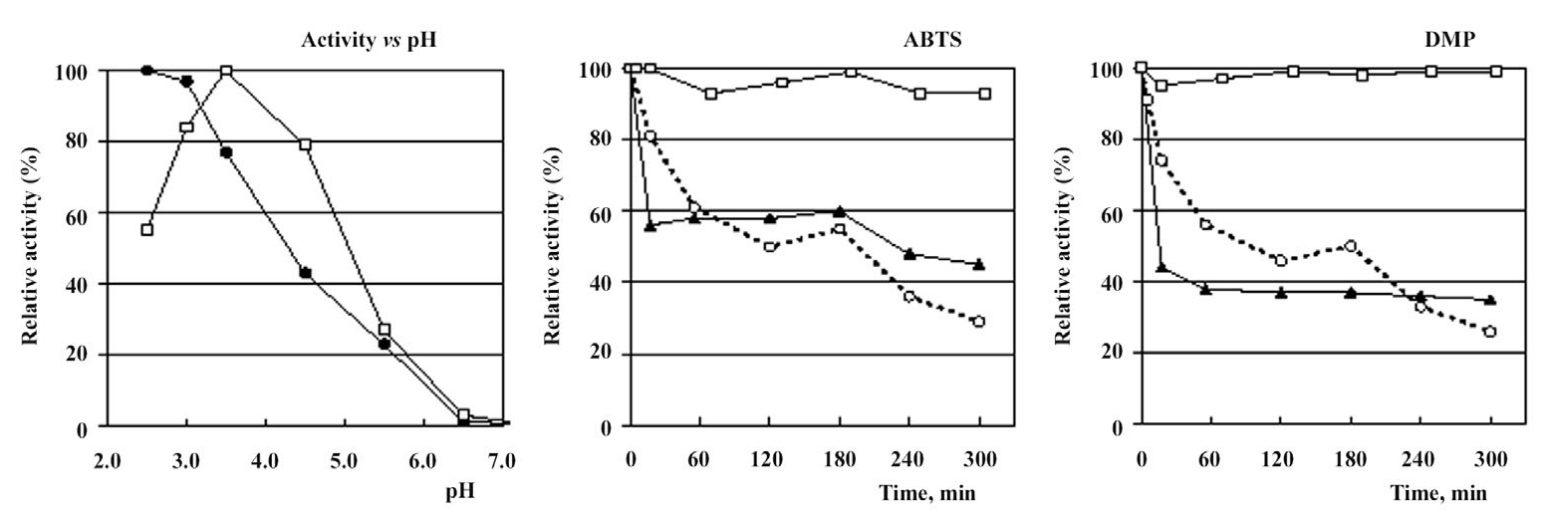
**Biochemical characterization of the recombinant Lcc1**. pH/activity profiles of recombinant Lcc1 with ABTS (●) and DMP(□) and Lcc1 stability at different pH at 25°C (○ pH 2.5, ▲ pH 3.5, □ pH 6.0). Data points are averages of triplicate measurements with SD within 10% of the mean.

The pH optima for the purified recombinant Lcc1 were determined with phenolic (DMP) and non phenolic (ABTS) substrates. The pH activity profiles (Fig. 3) were found to be very similar to those obtained for the native laccase from *T. trogii *[18]. A bell shaped profile with a maximum at pH 3.5 was observed when DMP was used as a substrate, while the optimal activity with ABTS was measured at the lowest limit of the pH range (pH 2.5).

The stability of the recombinant enzyme was studied at 25°C in McIlvaine buffer at three pH values: 2.5 (high activity *vs *ABTS), 3.5 (high activity *vs *phenolic substrates) and 6.0 (a pH value closer to the neutrality; Fig. 3). Lcc1 was stable at pH 6.0, since the activity decreased less than 10% after 5 h of incubation at pH 6.0, while at pH 2.5 and 3.5 the half-life was about 1 h and the residual activity after 5 h was about 40%. The thermostability experiments indicated that the enzyme at 60°C and pH 6.0 loses about 90% of its activity after 3 hours (data not shown).

Kinetic parameters (K_M_, k_cat_, K_A_) of the recombinant laccase on ABTS, DMP and guaiacol are shown in Table 2. The values obtained were in the same order of magnitude of those determined for the native laccase [18], with a k_cat_/K_M _at least 100-fold higher for ABTS than for DMP.

**Table 2:** Kinetic parameters of recombinant and native Lcc1

**Substrate**	**Recombinant Lcc1**			**Native Lcc1**		
	
	K_M _(μM)	k_cat _(min^-1^)	k_cat_/K_M _(μM^-1^min^-1^)	K_M _(μM)	k_cat _(min^-1^)	k_cat_/K_M _(μM^-1^min^-1^)
**ABTS**	9.2 ± 0.7	5899 ± 100	641	8.3 ± 0.3	5865 ± 55	715^a^
**2.6-DMP**	529 ± 27	3339 ± 55	6.31	195 ± 0.01	3064 ± 46	15.7^a^
**Guaiacol**	4177 ± 70	305 ± 3	0.07	3073 ± 88	392 ± 3	0.13^b^

^a ^(18); ^b ^this work.

### 5. Decolourization experiments and effect of organic solvents on Lcc1

**Figure 4:**
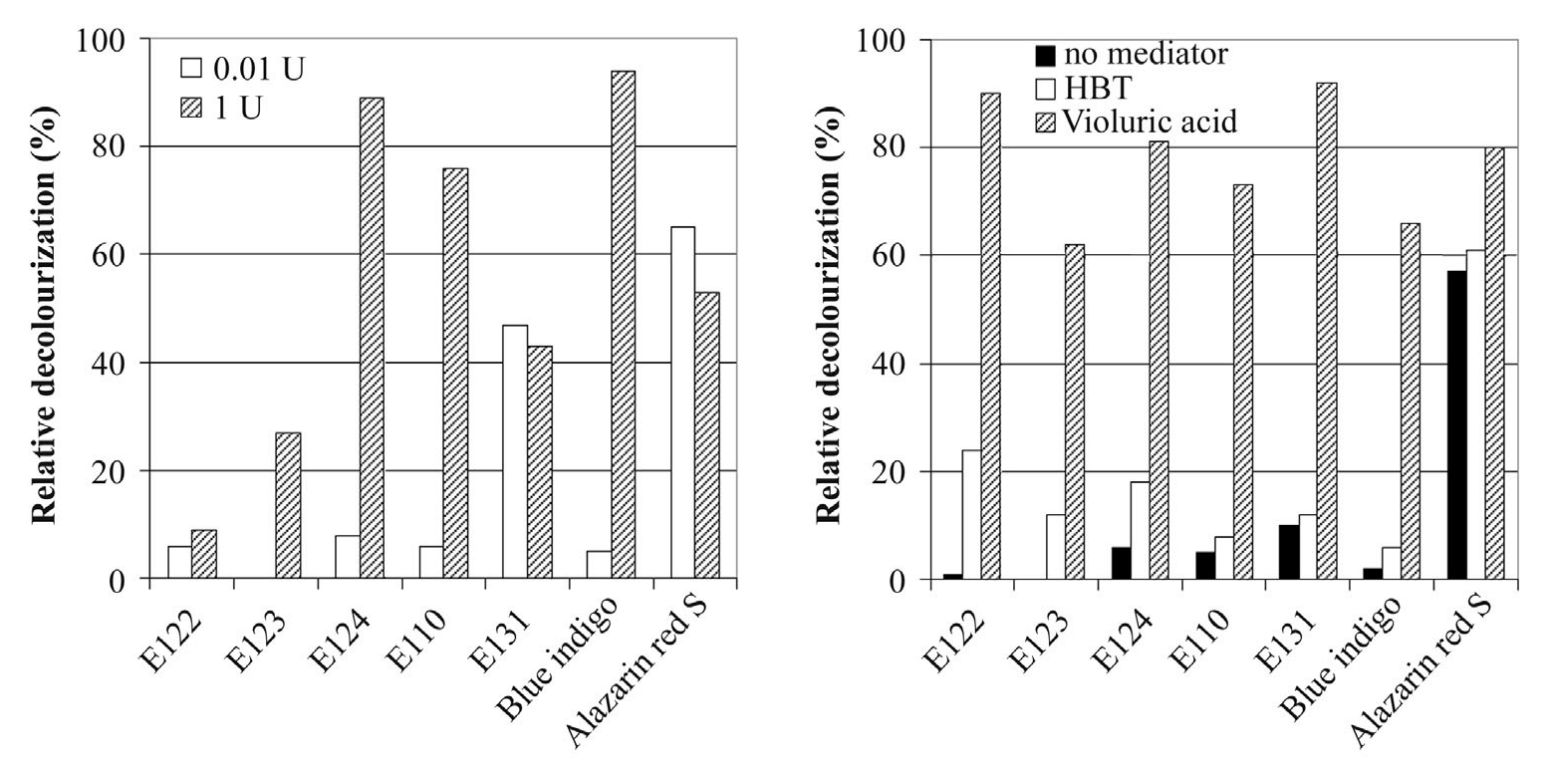
**Decolourization of dyes by recombinant laccase**. Left panel: decolourization percent after 6 hours in a reaction mix containing 0.01 U or 1 U of Lcc1; right panel: decolourization percent after 2 hours in a reaction mix containing 0.01 U of Lcc1 and redox mediators, HBT or violuric acid 1 mM. (E122, carmoisine; E123, amaranth; E124, cochineal red; E110, sunset yellow; E131, patented blue). Data points are averages of triplicate measurements with SD within 10% of the mean.

The recombinant enzyme has been tested for its ability to decolourize several synthetic dyes including azo dyes (amaranth, carmoisine, cochineal red, sunset yellow), triarylmethane (patented blue), indigo carmine and the anthraquinonic dye alizarin red S, in the presence and absence of redox mediators. Decolourization was followed with a spectrophotometer as the relative decrease of absorbance maximum of each dye. The tested dyes were decolourized by Lcc1 at a different extent, depending on the structure and complexity of each molecule (Fig. 4). By increasing the amount of enzyme in the reaction mixture, an increase of the decolourization percent was observed with indigo carmine and azo dyes, whereas the decolourization of patented blue and alizarin red S was only slightly affected; carmoisine and amaranth were the molecules more recalcitrant to degradation. In order to improve the enzymatic decolourization, the effect of the redox mediators 1-hydroxybenzotriazole and violuric acid was also tested (Fig. 4). In the presence of these compounds, an increase in the rate of decolourization of all the dyes was already observed after 2 hours and with the lowest amount of Lcc1 (0.01 U). Nevertheless, remarkable differences were observed between the two mediators: in the presence of violuric acid more than 60% of the colour was removed after 2 hours. After 6 hours of incubation the decolourization percent was in all cases higher than 80% and some of the differences between the mediators were less evident (data not shown).

We also evaluated the effects on the activity of recombinant laccase of water-miscible organic solvents (acetone, methanol and DMSO) that can be used for a variety of technical applications. The presence of organic solvents in moderate amount (up to 20% v/v) either increased (methanol) or did not affect significantly (acetone, DMSO) the laccase activity. Increasing the concentration of acetone or methanol in the assay mixture caused a moderate decrease of the laccase activity; DMSO at concentrations higher than 20% affected much more the enzyme activity. The recombinant laccase showed a fairly good stability in the presence of 20% acetone, methanol or DMSO: after 5 hours incubation, the residual laccase activity was about 90%. The behaviour of the recombinant laccase in the presence of organic solvents is comparable to that of the native enzyme (data not shown).

## Discussion

The *P. pastoris *expression system has been used for the production of a wide variety of proteins [8]. The expression of recombinant genes is under the control of the tightly regulated alcohol oxidase promoter (P_AOX1_), which is induced more than 1000-fold when cells are shifted to methanol as the sole carbon source; moreover, when grown on minimal medium, *P. pastoris *secretes recombinant proteins in the presence of low levels of native proteins, which facilitates the recovery and purification of the target protein [21]. There are a number of strategies used to optimize the production level of a heterologous protein in *P. pastoris*, which include the control of environmental parameters such as temperature, pH, methanol concentration, as well as the use of protease deficient strains in order to prevent the proteolytic degradation of the recombinant protein. Growing the yeast to high cell density enhances the production of recombinant proteins [22], but the pH of the culture medium must be carefully controlled in order to prevent any enzyme damage. Moreover, direct positive correlation between the amount of methanol consumed and the quantity of recombinant protein produced is observed [23].

Notoriously laccases, like other ligninolytic enzymes, have been rather difficult to produce in large amounts as recombinant proteins. Even if shaken flasks as a fermentation method are simple to handle and have a low cost, the culture volume was too low to be convenient for the production of the recombinant enzyme. Our results indicated that it was possible to enhance both the volumetric and specific laccase productivity in *P. pastoris *cultures increasing the amount of methanol added under carefully controlled conditions; the recombinant enzyme was quite stable in the presence of this solvent. As estimated from the specific activity of the purified enzyme, the amount of laccase produced by *P. pastoris *corresponds to about 17 mg per liter, a value which is two-three times higher than those obtained for other laccases [11,12]. Moreover, the amount of laccase produced using the recombinant system is higher than that obtained by *T. trogii *cultures which always produces a number of laccase isoforms that are difficult to separate having similar chemico-physical properties [19].

Native and yeast secretion signals could be used to direct the secretion of the enzyme, with the native signal yielding higher enzyme activity in the culture medium. The higher effectiveness of the native laccase signal peptide has been reported by Brown et al. [24] for the expression of an isozyme from *T. versicolor *by *P. pastoris*. The use of the *S. cerevisiae *α-mating factor signal peptide caused the retention of a tetrapeptide at the N-terminus of the recombinant protein; this modification was postulated to be responsible of the specific activity reduction of *T. versicolor *laccase [24]. As well, Jönsson et al. [9] demonstrated that the amount of *T. versicolor lcc1 *laccase produced in *P. pastoris *was seven-fold higher when the native secretion signal was used. Soden et al. [12] showed that the native signal peptide of *P. sajor-caju *Lac4 was effective in directing both the secretion and proper proteolytic maturation of the recombinant protein. In conclusion, the use of native laccase signal sequences from *Pleurotus *and *Trametes *species may be a viable alternative to the α-factor signal peptide to drive the secretion of recombinant proteins in *P. pastoris*.

The recombinant laccase Lcc1 was purified 89-fold, with a 16% yield, by a protocol similar to that used for the native enzyme from *T. trogii*; as expected, specific activities, kinetic properties, pH optimum for different substrates, stability to pH and solvents showed that Lcc1 from *P. pastoris *was similar to the main laccase from *T. trogii*.

In our study, we demonstrated that the recombinant laccase Lcc1 could be a useful biocatalyst for the oxidative degradation of several polluting dyes. We analysed the decolourization of seven dyes belonging to different structural groups and commonly used in industrial applications and we observed that the laccase preparation directly decolourized indigo carmine, antraquinonic, triarylmethane and a few azo dyes (Fig. 4).

Indigo carmine, the most important dye in the manufacturing of blue jeans, could be degraded by laccase yielding isatin (indole-2,3-dione) as an intermediate, which was further decomposed to give anthranilic acid (2-aminobenzoic acid) as a final reaction product [25]. Indigo carmine was almost completely and rapidly decolourized by Lcc1 using 1 U of enzyme without the addition of any redox mediator, which could be an important advantage for the future industrial usage of this enzyme. On the contrary, Wong and Yu [26] showed that indigo carmine was not a direct substrate for a laccase from *T. versicolor*, while an anthraquinonic dye was directly oxidized by the enzyme.

Not all the azo dyes were oxidized by Lcc1 at the same extent: carmoisine and amaranth were more recalcitrant to degradation than cochineal red and sunset yellow. The differences in azo dyes oxidation could be explained by the different electron donating properties of the substituents and their locations on the phenolic ring. It has been demonstrated that laccases preferably oxidized phenols with *ortho*-, *para*-oriented groups in *ortho *and/or *para *position [17]. This effect is more pronounced in cases of substituents with lone electron pairs because of their electron donating character, suggesting that only electron-rich phenolic rings can be oxidized by laccase. Chivukula and Renganathan [27] suggested a mechanism also for azo dyes oxidation by laccase, in which the enzymatic generation of a phenoxy radical results in the cleavage of azo linkages with the release of molecular nitrogen. The result was not only decolourization of the dye but also the exclusion of aromatic amine formation [28].

Phenolic and nonphenolic compounds with high E_0 _values can be oxidized by laccase through the mediation of small, redox active substrates like synthetic compounds of the NOH-type, as well as several lignin-derived phenolic compounds. Our results showed that the addition of a synthetic mediator significantly improved decolourization of all the dyes tested by the recombinant laccase Lcc1, and violuric acid was more efficient than 1-hydroxybenzotriazole. All the dyes were decolourized more efficiently and rapidly in the presence of redox mediators, using the lowest amount of the enzyme. Several workers found that the redox potential of fungal laccases varied depending on the source of the enzyme; this could dictate the need and/or nature of redox mediator for the degradation of a particular dye to occur [29].

Catalytic assays performed in the presence of organic solvents indicated that Lcc1 tolerates moderate amounts of organic solvents better than other fungal laccases, such as those from *T. versicolor *and *P. ostreatus *[30]. Enzymes are being used increasingly in non-conventional solvents mainly to improve solubility of certain substrates [31]. In the case of laccases, the presence of organic solvents is required for the oxidation of hydrophobic substrates such as the polycyclic aromatic hydrocarbons. Several studies evaluated a homogeneous aqueous-organic system for laccase catalyzed oxidative reduction of biphenyl derivatives and found that optimized systems had the potential to completely oxidize several hydroxy biphenyls [30,32].

## Conclusion

In this work, we demonstrated that the expression of *lcc1 *gene from *T. trogii *in the methylotrophic yeast *P. pastoris *produces an extracellular laccase which is stable and active in the presence of moderate amounts of organic solvents. This enzyme has a good decolourization capacity toward several synthetic dyes; this capacity can be enhanced by the addition of mediators such as violuric acid. The use of Lcc1 could be conceivably extended to other textile dyes and the scale up of the decolourization process could be worthy of further investigation.

## Methods

### 1. Organism and culture conditions

The *Pichia pastoris *strain GS115 (*his4*) was purchased from Invitrogen and yeast media and agar plates were prepared as described by the manufacturer. Inocula were prepared by transferring cells from minimal dextrose (MD) agar plates into 500 ml Erlenmeyer flasks containing 50 ml of phosphate buffered yeast nitrogen base supplemented with glycerol (2%) and biotin (400 μg/l). Cultures were grown at 30°C in an orbital shaker (150 rpm) and cells harvested in log-phase growth were used as inoculum for shake-flask and fermentor cultivations.

Shake-flask cultivations were performed at 30°C in phosphate buffered minimal methanol (BMM) supplemented with yeast extract (1%; BMMY) or casaminoacids (1%; BMMC). Cells harvested from the inoculum were directly resuspended in BMMY or BMMC medium to an OD_600 _of 1.0. Methanol was added daily to a final concentration of 0.5% to maintain induction.

### 2. Expression of lcc1 cDNA from T. trogii in P. pastoris

The cDNA of *lcc1 *from *T. trogii *was cloned under control of the methanol-inducible alcohol oxidase (*AOX1*) promoter of *P. pastoris *into the expression vectors pHIL-D2 and pPIC9 (Invitrogen). Two recombinant plasmids were obtained: pHIL-D2/*lcc1*, containing the *lcc1 *cDNA including the native signal sequence, and pPIC9/*lcc1*, in which the cDNA sequence encoding the native Lcc1 signal peptide was exchanged for that encoding the *Saccharomyces cerevisiae *α-mating factor signal peptide.

Plasmid DNA was digested with *Sst*I (pHIL-D2/*lcc1*) or *Stu*I (pPIC9/*lcc1*) prior to transformation for efficient integration into the *P. pastoris *genome. *P. pastoris *GS115 *(his4*) cells were transformed by electroporation with a GenePulser II apparatus (Bio-Rad Laboratories). Vectors without *lcc1 *cDNA were also used to prepare control strains. The electroporated cells were plated onto histidine-deficient MD agar plates and incubated at 30°C for 72 h, after which His^+ ^transformants were screened on minimal methanol (MM) agar plates containing the chromogenic compound ABTS which served as reducing substrate for the laccase. The activity was determined by the appearance of a green colour due to the oxidation of ABTS. More than 90% of the transformants were positive for laccase activity, while no positive clone was detected when GS115 cells were transformed with linearized vectors (pHIL-D2 or pPIC9) as a control.

### 3. Fermentor cultures

Fermentation media and trace salts (PMT1) were prepared as described by Invitrogen. A GS115(pHIL-D2/*lcc1*) transformant was grown in a 2-l fermentor (Applikon) at 25°C in basal salts medium containing glycerol as carbon source and PMT1 trace salts (4.8 g/l). Ammonium hydroxide was used to maintain a pH of 5.0 and served as nitrogen source for the fermentation. An inoculum of 50 ml was added to the fermentor in order to have a final optical density of 1.0 at 600 nm. After exhaustion of the glycerol, a 50% v/v glycerol feeding was carried out for 24 h, at a feed rate of 20 ml/l·h. After that, the culture was induced by replacing the glycerol feed with a methanol feed at 1 ml/l·h. The feed was gradually increased up to 6 ml/l·h and the fermentation was continued for a further 96 h. During the methanol fed-batch phase, the dissolved oxygen (DO) was kept at 30–35% by controlling manually agitation (from 750 to 1000 rpm), total air flow (from 1 to 1.5 v/v/min) and amount of pure oxygen (up to 0.2 v/v/min).

In optimized conditions the methanol feed reached the flow rate of 18 ml/l·h. Stepwise increases of the methanol feed rate of 1 ml/l·h were carried out at 1 hour intervals if there was no methanol accumulation in the medium. To verify this a "DO spike" was performed; a DO spike consists of shutting down the methanol feed pump and timing how long it takes for the dissolved oxygen to rise 10%; once the DO has increased 10%, the feed pump is turned back on. The feed rate was increased only if the spike time was less than 30 sec.

### 4. Enzyme purification

The cultural broth was clarified by filtration on 0.45 μm cut-off filters, concentrated by ultrafiltration on a cellulose membrane (cut-off 10 kDa, Millipore) at 4°C, and equilibrated in 10 mM imidazole/HCl buffer pH 6.0. The protein concentrate was fractionated by anionic exchange chromatography on a Q-Sepharose Fast Flow matrix (1.5 × 30 cm, Pharmacia); the column was equilibrated at a flow rate of 2 ml/min with 10 mM imidazole/HCl buffer pH 6.0, and the proteins were eluted with a NaCl gradient from 0 to 0.4 M in 350 ml, and from 0.4 to 2 M in 50 ml. The eluate was monitored for absorbance at 280 nm and laccase activity. The active fractions were pooled, concentrated by ultrafiltration and equilibrated in 10 mM imidazole/HCl buffer pH 6.0 containing ammonium sulphate 1.7 M. The sample was loaded on a hydrophobic interaction chromatographic column (1.5 × 15 cm, Phenyl Sepharose, Pharmacia) equilibrated at a flow rate of 2 ml/min with the same buffer containing ammonium sulphate 1.7 M, and eluted with a linear gradient of ammonium sulphate from 1.7 to 0 M (200 ml) in 10 mM imidazole/HCl buffer pH 6.0. The fractions with laccase activity were pooled, concentrated by ultrafiltration, loaded on a Superdex 75 column (1 × 30 cm, Pharmacia) and eluted with 10 mM imidazole/HCl buffer pH 6.0 at 0.5 ml/min flow rate. The active fractions were pooled and concentrated by ultrafiltration. The Superdex 75 column was calibrated with blue dextran (2000 kDa), bovine serum albumin (69.8 kDa), ovalbumin (49.4 kDa), chymotrypsinogen A (21.2 kDa) and ribonuclease A (15.8 kDa).

### 5. Electrophoresis and electrofocusing

Polyacrylamide (7.5%) slab gel electrophoresis in 0.1% sodium dodecyl sulphate (SDS-PAGE) was carried out as described by Laemmli [32]. The gels were calibrated with the Prestained SDS Marker kit (BioRad). Gel staining was performed with Coomassie Brilliant Blue R-250. Native PAGE was carried out on 7.5% polyacrylamide gels at pH 8.8 under non-denaturating conditions; laccase activity was visualized in the gel with *p*-phenylendiamine (10 mM) as substrate in 0.1 M acetate buffer pH 5.0.

### 6. Protein deglycosylation

Native and recombinant laccases were deglycosylated by treatment with EndoH_f _(New England Biolabs). The glycoproteins were denatured for 10 min at 100°C in denaturing buffer (0.5% SDS and 1% β-mercaptoethanol) and then incubated overnight at 37°C with 1000 IU of EndoH_f _in 50 mM sodium citrate buffer pH 5.5. The reaction mixture was then submitted to SDS-PAGE; carbohydrate staining was performed by dipping gels in 12.5% trichloroacetic acid for 90 min, then in 1% periodic acid, 3% acetic acid for 1 h, washing with water, and incubating overnight in the Schiff reagent.

### 7. Protein and enzyme activity determinations

The protein concentration was determined using the Coomassie Plus Protein Assay Reagent (Pierce), with bovine serum albumin as standard, following the manufacturer instructions.

The automatic analysis of the N-terminal sequence of the purified Lcc1 was carried out at CEINGE, Advanced Biotechnologies, Department of Organic Chemistry and Biochemistry, University of Napoli, Italy.

Spectrophotometric assays of laccase activity were carried out at 25°C with 2 mM 2.2'-azino-bis-(3-ethylbenz-thiazolinesulphonate) (ABTS), 10 mM 2.6-dimethoxyphenol (DMP) or 20 mM 2-methoxyphenol (guaiacol) as substrates, in 0.1 M citric acid/0.2 M K_2_HPO_4 _(McIlvaine) buffer pH 3.4, in a final assay volume of 1 ml. ABTS oxidation was monitored at 420 nm (ε_mM _= 36.0 mM^-1^cm^-1^), DMP oxidation at 468 nm (ε_mM _= 27.5 mM^-1^cm^-1^) and guaiacol oxidation at 470 nm (ε_mM _= 26.6 mM^-1^cm^-1^); the enzymatic activity was expressed as IU.

In order to check the presence of intracellular laccase activity, the cells were lysed in a breaking buffer (50 mM sodium phosphate pH 7.4, 1 mM phenylmethylsulfonyl fluoride, 1 mM EDTA, 5% glycerol) using acid washed glass beads (0.5 mm). The mixture was vortexed for 30 seconds and cooled in ice for 30 seconds for 10 times, and then centrifuged at 12000 g for 10 minutes. The clear supernatant was transferred in a fresh container and used for the activity assay.

The Michaelis constants (K_M_) were obtained by assaying laccase activity in a range of substrate concentration from K_M_/4 to 4 K_M_; an approximate evaluation of K_M _was derived from a direct linear plot. K_M _and V_max _values were calculated by the nonlinear regression MacCurveFit (Kevin Raner Software, version 1.3); k_cat _(V_max_/[E]_tot_) and K_A _(k_cat_/K_M_) were then derived.

The activity/pH profiles of laccases were determined in the McIlvaine buffer with different ratios of 0.1 M citric acid and 0.2 M K_2_HPO_4 _to reach pH values in the range 2.5–7.5.

The activity of laccase in organic solvents was analysed with DMP as substrate in a mixture containing McIlvaine buffer and methanol, acetone or dimethyl sulfoxide (DMSO) at different concentrations (10, 20, 30% v/v). The stability was tested incubating the enzyme with 20% organic solvent for 5 hours.

### 8. Decolourization experiments

Dyes representing different chemical classes were purchased from Sigma-Aldrich: carmoisine (E122) λ_max _= 515 nm, amaranth (E123) λ_max _= 521 nm, cochineal red (E124) λ_max _= 506 nm, sunset yellow (E110) λ_max _= 480 nm, patented blue (E131) λ_max _= 625 nm, blue indigo carmine λ_max _= 610 nm, alizarin red S λ_max _= 423 nm. Stock solutions (0.5% in water) were stored in the dark at 4°C. The enzymatic treatment of textile dyes was performed in a reaction volume of 1 ml containing 0.05 mg/ml of dye in sodium phosphate buffer (0.1 M, pH 5.0) and 0.01 or 1 IU of recombinant laccase in the presence or absence of 1 mM redox mediator 1-hydroxybenzotriazole or violuric acid. Samples were incubated at room temperature and decolourization activity was determined on a spectrophotometer as the relative decrease of absorbance at the absorbance maximum of each dye. Results are the averages of three independent assays with SD within 10% of the mean.

## Authors' contributions

MCC participated in the design of the study, carried out the experimental work concerning plasmid and strain construction, yeast cultivation, decolourization experiments, and participated in drafting the manuscript. SL ran and analysed the fed batch fermentation. AMG participated in the design of this study and carried out the biochemical characterization and kinetic analysis of the recombinant enzyme. MR and VB participated in design and coordination of experiments, in data interpretation and drafted the manuscript. All authors read and approved the final manuscript.
